# Bone Remodeling Process Based on Hydrostatic and Deviatoric Strain Mechano-Sensing

**DOI:** 10.3390/biomimetics7020059

**Published:** 2022-05-06

**Authors:** Natalia Branecka, Mustafa Erden Yildizdag, Alessandro Ciallella, Ivan Giorgio

**Affiliations:** 1 Faculty of Mechanical and Industrial Engineering, Warsaw University of Technology, 00-661 Warsaw, Poland; natalia.branecka.dokt@pw.edu.pl; 2 International Research Center for the Mathematics and Mechanics of Complex Systems, University of L’Aquila, 67100 L’Aquila, Italy; yildizdag@itu.edu.tr (M.E.Y.); alessandro.ciallella@univaq.it (A.C.); 3 Faculty of Naval Architecture and Ocean Engineering, Istanbul Technical University, Istanbul 34469, Turkey; 4 Dipartimento di Ingegneria Civile, Edile-Architettura e Ambientale (DICEAA), University of L’Aquila, 67100 L’Aquila, Italy

**Keywords:** bone remodeling, mechanical stimuli, mechano-sensing, strain energy density, hydrostatic strain, deviatoric strain

## Abstract

A macroscopic continuum model intended to provide predictions for the remodeling process occurring in bone tissue is proposed. Specifically, we consider a formulation in which two characteristic stiffnesses, namely the bulk and shear moduli, evolve independently to adapt the hydrostatic and deviatoric response of the bone tissue to environmental changes. The formulation is deliberately simplified, aiming at constituting a preliminary step toward a more comprehensive modeling approach. The evolutive process for describing the functional adaptation of the two stiffnesses is proposed based on an energetic argument. Numerical experiments reveal that it is possible to model the bone remodeling process with a different evolution for more than one material parameter, as usually done. Moreover, the results motivate further investigations into the subject.

## 1. Introduction

The most important tasks of the skeleton include the protection of internal organs, supporting the entire body, and acting as the leveraging organ that enables mobility. Its main component is bone tissue, a type of hard connective tissue. The shaft of the long bones and the exterior layers of the flat bones constitute the cortical bone that carries the mechanical loads for the most part. The internal tissue of skeletal bone is called trabecular bone, and it is much more porous and flexible. Bone adapts to environmental conditions responding to changes in mechanical and biochemical stimuli. In this paper, we present a mathematical model whose task is to describe the influence of mechanical loads on bone remodeling, taking into account the interplay of the biological and mechanical reactions of tissue at a macroscopic level of description.

**Figure 1:**
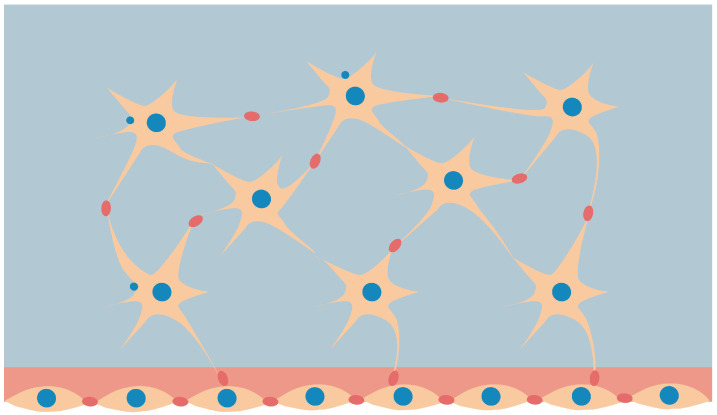
Osteocytes in bone matrix (star-shaped) with the formed OLCN connected to osteoblasts on the boundary.

Bone continuously adjusts to external mechanical loads, changing its properties through to the remodeling process. Bone adaptation is based on the sensing of mechanical loads and the continuous reconstruction of the bone architecture through the formation and resorption of bone tissue in a process that is influenced by external loads [1]. Mechanotransduction is the action of converting mechanical responses provided by external interactions into biochemical signals, which may induce the response of bone cells responsible for the synthesis or resorption of bone tissue. The cells in charge of this process of transducing, called osteocytes, are identified to be mechano-sensors, which detect mechanical signals and initiate and control the process of remodeling [2,3,4]. Osteocytes are regularly embedded in cavities of the bone matrix in hollow lacunae linked by narrow canals (see Figure 1). They form a network called the lacuno-canalicular network (OLCN) of processes that allows them to communicate with each other [5,6,7,8,9].

Interconnected cells can communicate via gap junctions, which permit the diffusion of ions, metabolites, and small signaling molecules from cell to cell. Gap junctions are located in the cell membranes of all bone cells, especially in actor cells, osteocytes, and osteoblasts and at the tip of osteocyte dendritic processes [2,10].

Mechanosensation of osteocytes includes a few types of loading, above all whole tissue strain, hydrostatic pressure, and streaming potentials generated by bone fluid flow through a charged bone matrix [4]. Owing to in vitro experiments, it is known that osteocytes are much more sensitive to fluid shear stresses than substantial fluid pressure [7]. The osteocyte is derived from the osteoblast, but osteocytes are much more responsive and more sensitive to shear stress [11,12,13]. When bone is under mechanical loading, the deformation of the bone matrix generates fluid flow in the OLCN, which induces shear stress along osteocyte membranes [14,15,16]. Due to the different sensitivity of sensor cells to pressure and shear stress, it seems more realistic to distinguish these processes in mathematical modeling. As a matter of fact, the mechanisms involved in the sensing function of the bone tissue mechanical state are distinct and located in different sites of the osteocytes; therefore, considering them in the same way is excessively simplistic.

The role of osteocytes is to send a proper signal to activate precursor cells [5], which will trigger the generation, as required, of actor cells, that is osteoblasts or osteoclasts. Osteoblasts and osteoclasts are bone cells directly responsible for bone remodeling in the case of microdamage, replacement of old bone with new bone, and bone adaptation [17,18,19,20]. Osteoblasts are adapted to cause bone formation and osteoclasts for bone resorption; it is hypothesized that they collect different types of biochemical signals, and their activity depends on the intensity. The entire process related to intercellular communication is not fully explained and needs ulterior investigations.

There are many theoretical models based on simplified assumptions. The problem of bone adaptation and remodeling has been considered since Wolff’s observations in 1892 that mathematical laws can describe changes in the architecture of bones [21,22]. Many mathematical models have been developed [23,24,25,26,27,28,29] dependent on various mechanical stimuli inducing bone adaptation. Among them can be mentioned diverse instances based on strain energy density [30,31], tissue damage [32,33,34], daily stress stimulus [35], effective stress [36], and strain [37]. In this paper, we follow the path of numerical models, where the assumption is made that bone mass adjusts in response to energy considerations, in which the loading history and energy transfers can be included [38,39]. The strain energy density (SED) is used as a control variable enabling defining the shape or bone density adaptations due to functional requirements [39,40]. A slightly different approach can be found in papers [40,41,42,43,44,45,46], where the classical proportional integral derivative (PID) control was employed to describe the mass density optimization macroscopically due to the remodeling process based on the feedback variable SED. Herein, we generalize the description of the bone evolution, by including two evolutive laws on the bulk and shear moduli, in order to focus on the bone remodeling responses arising from hydrostatic and deviatoric loadings. The main idea behind the paper is to explore the simplified case of isotropic bone tissue as a preliminary step to move forward with the more realistic case of anisotropic or orthotropic tissue. In these last cases, we have to deal with many material parameters; therefore, understanding what occurs in the case of two evolving material parameters appears crucial in developing a helpful model for accurately describing the remodeling process.

## 2. Materials and Methods

In this study, for the sake of simplicity, bone is assumed to be an isotropic elastic material. We can formulate Hooke’s law to define the stress tensor $\sigma_{ij}$ as a linear combination of the volumetric and deviatoric strain tensors:

(1) $$\sigma_{ij}=3K\left(\frac{1}{3}\varepsilon_{kk}\delta_{ij}\right)+2G\left(\varepsilon_{ij}-\frac{1}{3}\varepsilon_{kk}\delta_{ij}\right)$$

where *K* is the bulk modulus and *G* is the shear modulus. From Equation (1), it is possible then to recognize the two contributions to the stress, namely the hydrostatic:

(2) $${\sigma^{h}}_{ij}=3K\left(\frac{1}{3}\varepsilon_{kk}\delta_{ij}\right)$$

and shear or deviatoric part:

(3) $${\sigma^{s}}_{ij}=2G\left(\varepsilon_{ij}-\frac{1}{3}\varepsilon_{kk}\delta_{ij}\right)$$

This formulation can be generalized by using mechanical micromorphic [47,48,49,50,51,52,53,54,55,56], micropolar [57,58,59,60], higher-order [61,62,63,64,65,66,67,68,69,70], or peridynamic [71,72,73,74] models. As an opening move, the stiffnesses can be evaluated starting with the knowledge of the engineering parameters, Young’s modulus *Y*, and Poisson’s ratio ν as follows:

(4) $$K=\frac{Y}{3(1-2\nu )}$$

(5) $$G=\frac{Y}{2(1+\nu )}$$

since they are more straightforward to retrieve in the literature. The components of the strain tensor, in the linear approximation, can be written as:

(6) $$\varepsilon_{ij}=\frac{1}{2}\left(u_{i,j}+u_{j,i}\right)$$

The index after the comma stands for the differentiation with respect to the corresponding space variable. Based on that, we can find the equations describing the strain energy density decomposed into a component associated with the hydrostatic part of the load and deviatoric one:

(7) $$W=W_{h}+W_{d}$$

We remark that the decomposition of the energy in these two portions is quite convenient to use in the evolution of the stiffnesses since they are energetically orthogonal. As a matter of fact, they are devoid of any mutual coupling. For our investigation, we consider a 2D case, and specifically, the hydrostatic contribution is

(8) $$W_{h}=\frac{1}{2}K{\left(\varepsilon_{11}+\varepsilon_{22}\right)}^{2}$$

while the deviatoric part is

(9) $$W_{d}=G\left[\varepsilon_{11}^{2}+\varepsilon_{22}^{2}+2\varepsilon_{12}^{2}-\frac{1}{2}{\left(\varepsilon_{11}+\varepsilon_{22}\right)}^{2}\right]$$

We implement this way of representing the strain energy density for the problem of mechanically loaded bone. The bodies in charge of sensing mechanical loads, i.e., osteocytes, are assumed to be regularly distributed in the tissue volume. This simplifying hypothesis can be easily generalized by introducing an inhomogeneous density of osteocytes (see, e.g., [31]). However, in this work, we refrain from introducing this feature since, locally, we can safely assume that the change in the number of osteocytes is not quickly variable in the space. Osteocytes transform a mechanical signal into a biochemical one, which is, in our simplified model, instantaneously transmitted, and its intensity decays exponentially as the distance from the osteocytes (the source of the signal) increases [31,75,76]. Therefore, we consider an influence radius surrounding the osteocytes, i.e., *D*, and in our example, we set for it the value 0.1 mm. In the 2D example, we can write the normalized distance from a sensor cell, thought to be located in $X_{0}$, to a given material particle in which the actor cells, namely osteoclasts and osteoblasts, act as:

(10) $$E_{s}=\frac{∥X-X_{0}∥}{D}$$

The biochemical signal transmitted by osteocytes triggers the activities of the actor cells, i.e., osteoblasts and osteoclasts, in the process of bone adaptation to mechanical stimulation. According to the previous partition into hydrostatic and deviatoric parts, we can postulate the biochemical stimuli to be:

(11) $$S_{K}\left(X,t\right)=\int_{\Omega }W_{h}\left(X_{0},t\right)e^{-E_{s}\left(X,X_{0}\right)}dX_{0}-S_{K0}\left(X,t\right)$$

(12) $$S_{G}\left(X,t\right)=\int_{\Omega }W_{d}\left(X_{0},t\right)e^{-E_{s}\left(X,X_{0}\right)}dX_{0}-S_{G0}\left(X,t\right)$$

The osteocytes are mechanoreceptor, i.e., sensing components that monitor and respond to mechanical changes in the environment. In particular, we assume that osteocytes transmit a biochemical signal that is proportional to the elemental strain energy of the volume particle in which they are located. When formulating the evolutionary equations, we link the change in material parameters *K* and *G* occurring in time with the signal transmitted to a given elementary volume of the material. The two quantities $S_{K0}$ and $S_{G0}$ represent the optimal functioning conditions from a mechanical viewpoint of the bone tissue. They define homeostasis, which is the state of steady internal, mechanical conditions that should be maintained by the living system, i.e., bone tissue, to guarantee its correct functionality. In other words, homeostasis is determined by a natural resistance to change when the biological system is already in the optimal condition. The primary purpose of the bone tissue is of a mechanical nature, and it is well known that the strength of materials is differently affected by hydrostatic and deviatoric deformations or stress. One could specifically think of the von Mises yield criterion based on maximum distortion energy attainable in the material, namely the maximal deviatoric part of the deformation/stress. This simple observation leads us to think that, in principle, these two quantities $S_{K0}$ and $S_{G0}$ are, in fact, different. Moreover, thinking about this functional aspect, it is no coincidence that the deviatoric part, related to $S_{G0}$, has a predominant role.

In this formulation, since the density of osteocytes is assumed to be uniformly distributed over the considered domain, we do not introduce a function representing the number of sensor cells per unit volume as a multiplicative factor to the energy density for the sake of simplicity, as done in [31] instead. In detail, the evolution laws for the two stiffnesses are assumed to be:

(13) $$\frac{\partial K(S_{K},t)}{\partial t}=A_{K}\left(S_{K}\right)H\left(\frac{K}{K_{\mathrm{Max}}}\right)$$

(14) $$\frac{\partial G(S_{G},t)}{\partial t}=A_{G}\left(S_{G}\right)H\left(\frac{G}{G_{\mathrm{Max}}}\right)$$

where the functions $A_{K}$ and $A_{G}$ are piecewise linear functions of the stimuli (11) and (12) as specified below:

(15) $$A_{K}\left(S_{K}\right)=\left\{\begin{array}{c} r_{sK}\;S_{K}\;\mathrm{for}\;\;S_{K}⩾0\\ r_{rK}\;S_{K}\;\mathrm{for}\;\;S_{K}<0 \end{array}\right.$$

and

(16) $$A_{G}\left(S_{G}\right)=\left\{\begin{array}{c} r_{sG}\;S_{G}\;\mathrm{for}\;\;S_{G}⩾0\\ r_{rG}\;S_{G}\;\mathrm{for}\;\;S_{G}<0 \end{array}\right.$$

The function H(x) is a weight for numerical calculation that prevents the complete vanishing, as well as unlimited growth of parameters *K* and *G* and is considered to be:

(17) H(x)=4x(1−x)

in the interval [0,1], while it is set to zero outside. The limit coefficients $K_{\mathrm{Max}}$ and $G_{\mathrm{Max}}$ represent the maximum values attainable for *K* and *G*, respectively.

**Figure 2:**
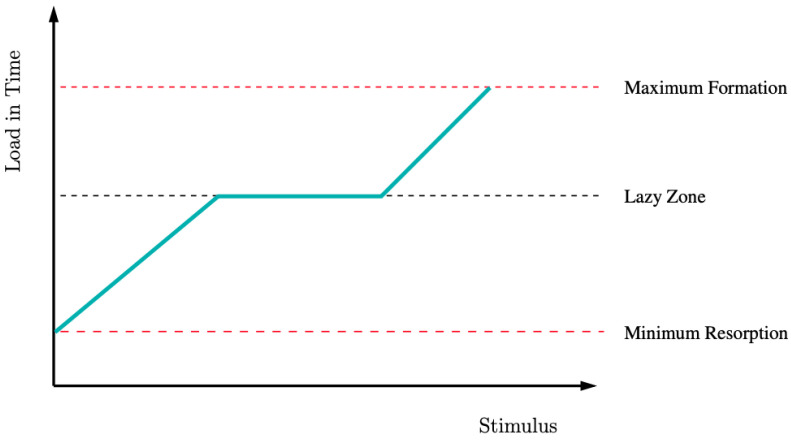
Effect of the load on stimulus. Low mechanical loading results in bone resorption: it is the resorption stimulus zone; subsequently, in the lazy zone (black dotted line), there is no change in bone; with a sufficiently high load, the formation of the stimulus zone occurs in a certain range highlighted with red dotted lines.

When the signal is within a certain range, even in the presence of a stimulus beyond the threshold, there is no actor cell activity [31] (see Figure 2). Ratios $r_{sK}$, $r_{rK}$, $r_{sG}$, and $r_{rG}$ are determined experimentally and are regulating factors. Ratio $r_{sK}$ scales the function $S_{K}$ when it is greater than or equal to 0; ratio $r_{rK}$ scales the function $S_{K}$ when it is smaller than 0, similar in the case of $r_{sG}$, which scales the function $S_{G}$ when it is greater than or equal to 0; ratio $r_{rK}$ scales the function $S_{K}$ when it is smaller than 0. The bone remodeling process depends on mechanical stimulus intensity: when it is too low, bone is reabsorbed; when it is too high, it causes damage, but there are also optimal zones where the bone is adapting its properties to external mechanical conditions [30,40].

In line with the assumption that strain energy is divided into the hydrostatic and deviatoric parts, in what follows, we can observe the impact that different kinds of mechanical loading have on bone remodeling through several numerical simulations.

## 3. Results

**Figure 3:**
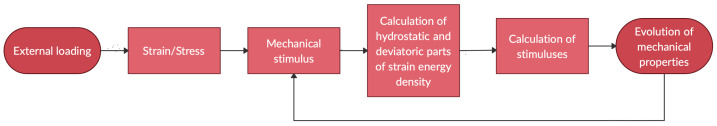
Flow chart of processes.

The numerical computations were performed on a 2D isotropic material with the initial values presented in Table 1. By following the schema of the process shown in Figure 3, an FE algorithm was written in Comsol Multiphysics in order to test and support the hypotheses behind our model, showcasing some plausible responses that can be compared at least preliminarily and qualitatively with real-life evolutions.

To illustrate the main feature of the proposed model, we simulated the evolution of several representative cases: two examples in which the external loads activate a pure hydrostatic and pure shear deformation, then a third case, where the two kinds of deformation are simultaneously present.

**Figure 4:**
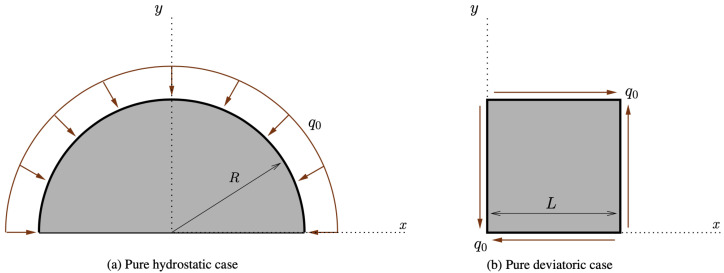
Schematics for the considered pure hydrostatic and pure deviatoric cases.

At first, we implemented the pure hydrostatic case, where a semicircular piece of bone, attached to the ground to avoid any rigid motion, but free to deform, was subjected to a distributed force perpendicular to its circular boundary (see Figure 4a).

**Figure 5:**
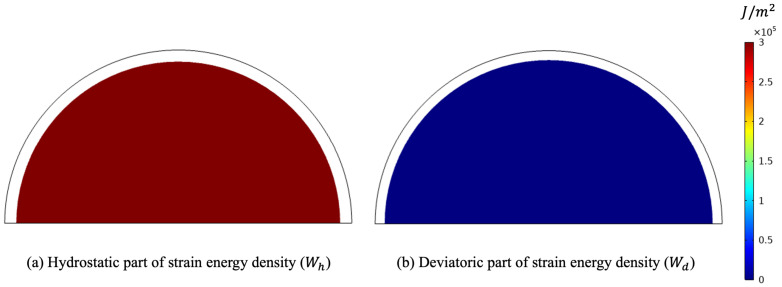
Deformed shape for the pure hydrostatic case (plots were obtained with a scale factor of 20).

In particular, the maximum diameter is linked to the ground with a perfect constraint in the vertical direction and with a weak elastic potential in the other direction to prevent rigid motion horizontally and allow deformation in that direction. Figure 5 clearly exhibits the orthogonal feature of the two energy contributions. As a matter of fact, due to the circular symmetry of the external load, also the deformation keeps this symmetry. As a consequence, no deformation involving a change of shape is activated.

**Figure 6:**
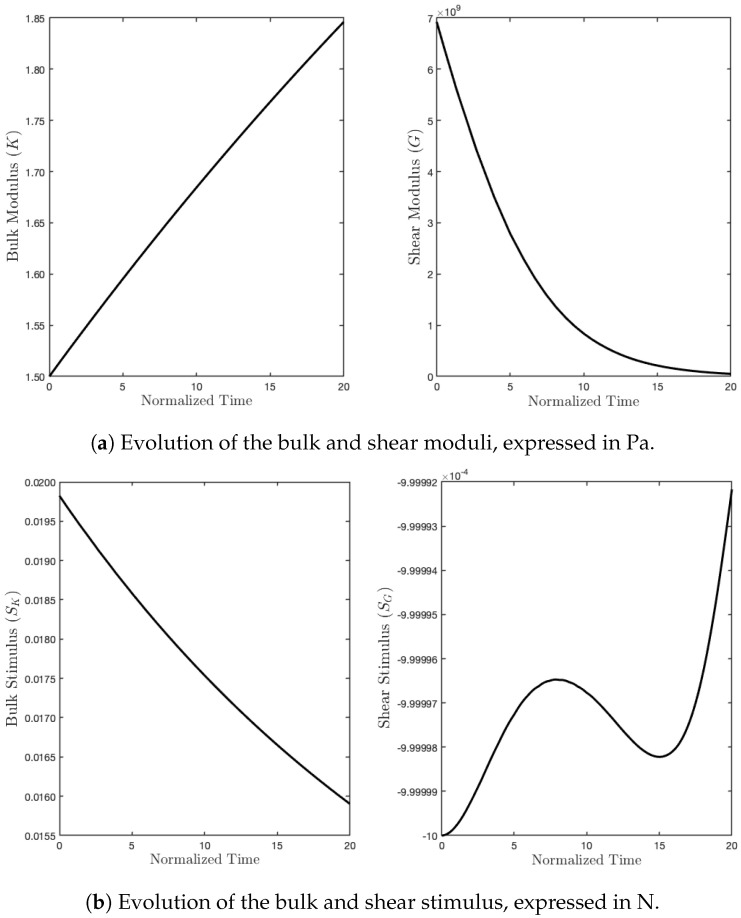
Evolution of moduli and stimuli in time for the purely hydrostatic case.

As we expected, the parameter *K*, during the evolution subject to such a mechanical load, denoted by $q_{0}$, increased due to a sufficiently high level of external action, which, in turn, produced a positive stimulus for this parameter. On the contrary, since the energy contribution of the deviatoric part is null, the related stimulus was negative, and therefore, the shear modulus started to fade (see Figure 6).

**Figure 7:**
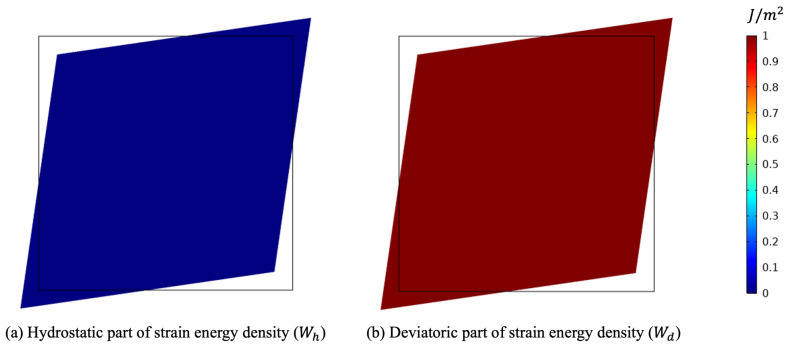
Deformed shape for the pure deviatoric case (plots were obtained with a scale factor of 20).

**Figure 8:**
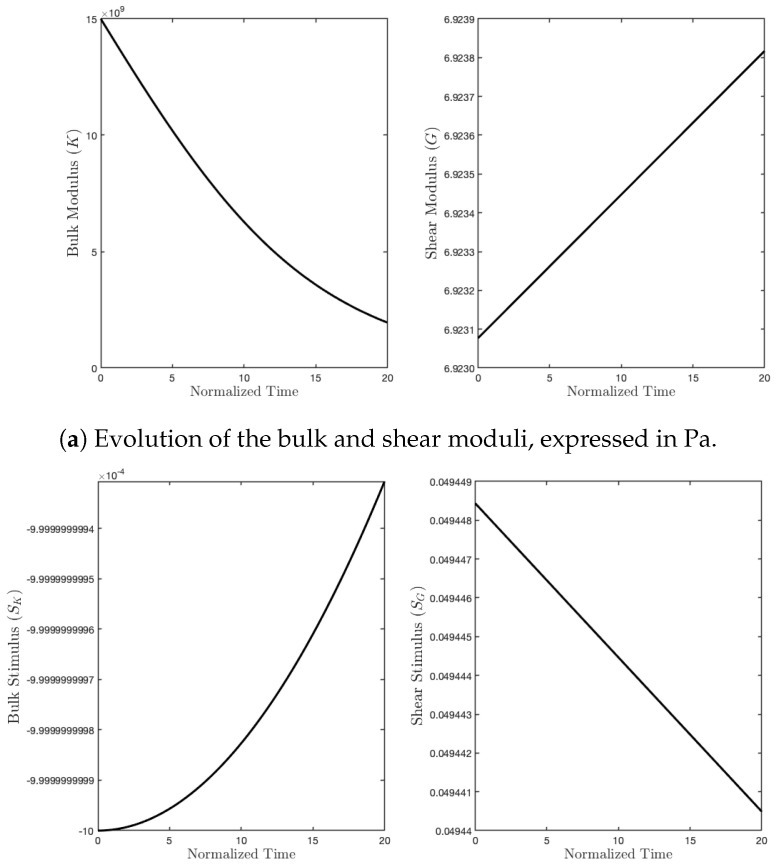
Evolution of moduli and stimuli in time for the purely deviatoric case.

Subsequently, we simulated the pure shear strain example (see Figure 4b). In this case, the external actions were conceived of to impose a deformation with a change of shape, but not of the area (see Figure 7). In contrast with the previous case, now, the two parameters *G* and *K* exchanged their role (see Figure 8). Indeed, *K* decreased because the related stimulus became negative (no hydrostatic energy was activated), while *G* increased for the external action, denoted by $q_{0}$, producing a positive stimulus.

Naturally, the evolution of *K* and *G* depends on the ratios $r_{sK}$, $r_{rK}$, $r_{sG}$, and $r_{rG}$, which are to be determined by comparison with experimental tests on living bone tissues.

**Figure 9:**
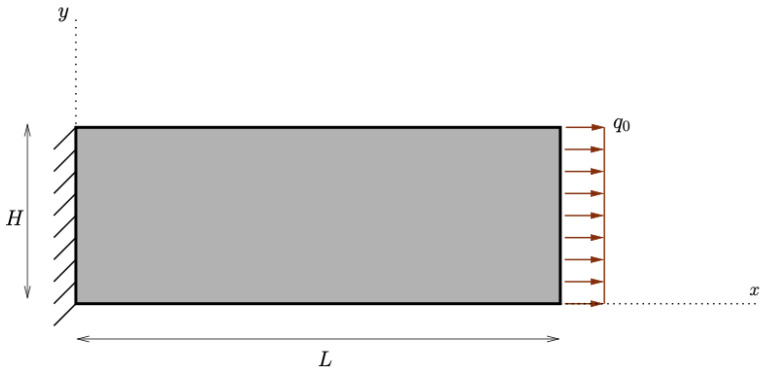
Schematic for the tensile test under a uniform load $q_{0}$.

**Figure 10:**
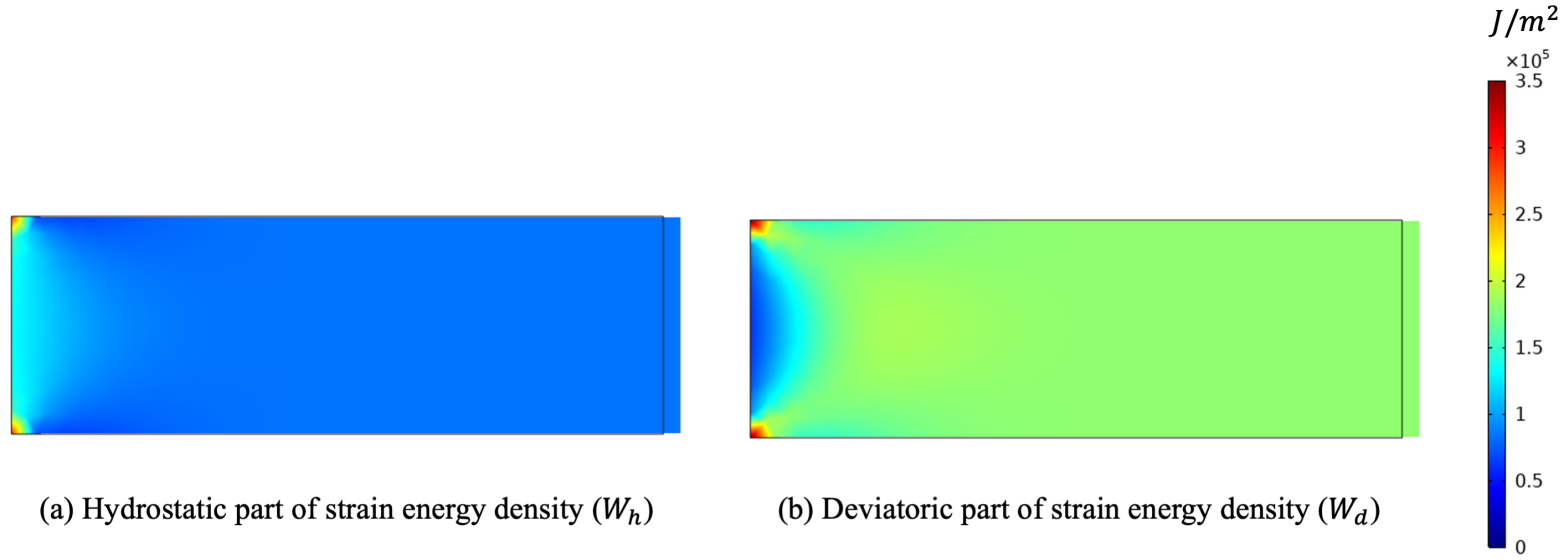
Deformed shape for the tensile test (plots were obtained with a scale factor of 5).

**Figure 11:**
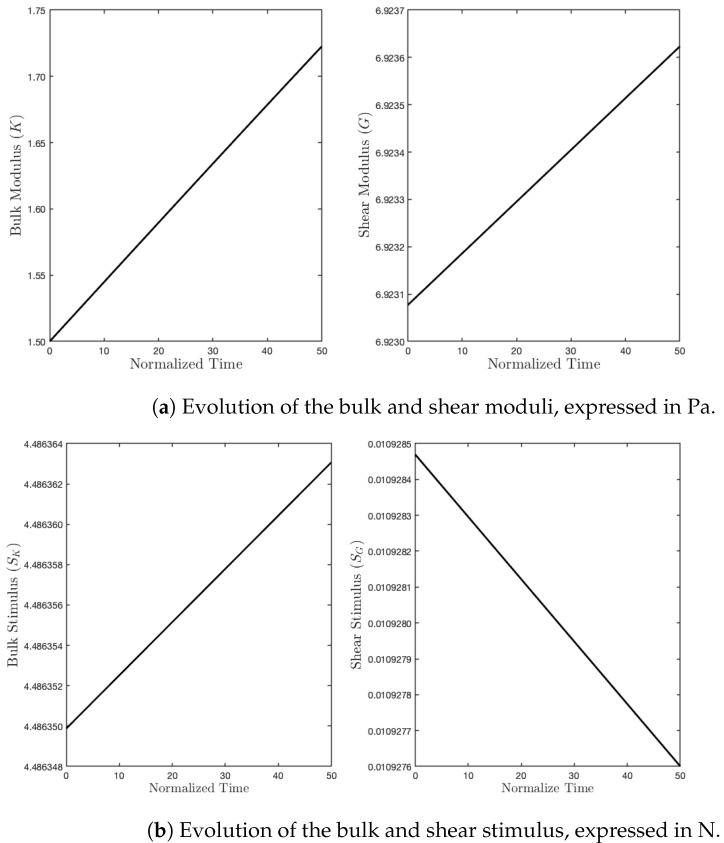
Evolution of moduli and stimuli in time for the tensile test.

Finally, we performed one simulation with a tensile test (see Figure 9). In this last example, both contributions of the energy were activated (see Figure 10). Here, both stimuli turned out to be positive, and hence, both stiffness parameters increased (see Figure 11).

The two kinds of deformations are almost always present in real applications, but their effects are expected to produce different outcomes. Indeed, due to the diverse nature of the material parameters involved in the evolution, one could associate their changes with various aspects. For instance, the modification in the apparent mass density is directly linked with the bulk stiffness *K* since a change in the porosity has a relevant effect on the hydrostatic response of the bone tissue (see, for a similar line of reasoning, the Appendix of [77]). On the other hand, the shear stiffness *G* is more responsible for the mechanical capability of the bone tissue to resist distortion deformations; therefore, a change in it will result in a different strength of the tissue associated with this type of deformation.

## 4. Conclusions

The main goal of this article was to develop a mathematical model that adequately describes the influence of hydrostatic and deviatoric loads on bone adaptation. The presented numerical simulations confirmed that the formulated mathematical description makes it possible to determine changes in bone stiffnesses, taking into account the nature of the different mechanical stimuli. It is well known that the influence of the deviatoric part is much more significant than the influence of the hydrostatic part on the magnitude of the transmitted signal and its impact on the actual bone transformation. The proposed model is able to capture this feature quite easily by changing independently a few material parameters that are responsible for the evolution of the mechanical stiffnesses, namely the ratios of the changing of the two parameters considered. This is in line with the knowledge to date regarding the function of osteocytes, which are most sensitive to flow shear loads and less to pure compression.

In future works, we plan to generalize the proposed approach to the case of orthotropic materials by introducing suitable stimuli for the evolution of each stiffnesses of the material. Indeed, the orthotropic hypothesis is more accurate in describing the mechanical behavior of a larger class of bone tissues [78,79]. However, in this work, we simplified the formulation because we believe that, especially when complex systems are to be studied, a practical approach is to explore different aspects involved in the phenomenon separately to understand their nature and develop an accurate model putting together all the insights obtained in the intermediate steps.

## Figures and Tables

**Table 1:** Parameters used in the numerical simulations.

Parameter	Value
$Y_{0}$	18 GPa
$\nu_{0}$	0.3
D	0.1 mm
$K_{\mathrm{Max}}$	30 GPa
$G_{\mathrm{Max}}$	13.84 GPa
$S_{K0}$	0.001 N
$S_{G0}$	0.001 N
$r_{sK}$	$1\times 10^{7}$ m${}^{-3}$s${}^{-1}$
$r_{rK}$	$1\times 10^{9}$ m${}^{-3}$s${}^{-1}$
$r_{sG}$	$1\times 10^{3}$ m${}^{-3}$s${}^{-1}$
$r_{rG}$	$1\times 10^{9}$ m${}^{-3}$s${}^{-1}$

## Data Availability

Not applicable.
